# Rejection sensitivity as a vulnerability marker for depressive symptom deterioration in men

**DOI:** 10.1371/journal.pone.0185802

**Published:** 2017-10-19

**Authors:** Jannika De Rubeis, Ricardo G. Lugo, Michael Witthöft, Stefan Sütterlin, Markus R. Pawelzik, Claus Vögele

**Affiliations:** 1 Institute for Health and Behaviour, Research Unit INSIDE, University of Luxembourg, Esch-sur-Alzette, Luxembourg; 2 Department of Psychology, Inland Norway University of Applied Sciences, Lillehammer, Norway; 3 Department of Clinical Psychology, Psychotherapy and Experimental Psychopathology, University of Mainz, Mainz, Germany; 4 Faculty for Health and Welfare Sciences, Østfold University College, Fredrikstad, Norway; 5 Center for Clinical Neuroscience, Division of Clinical Neuroscience, Oslo University Hospital, Oslo, Norway; 6 Eos-Klinik für Psychotherapie, Alexianer GmbH, Münster, Germany; 7 Research Group on Health Psychology, University of Leuven, Leuven, Belgium; Peking University, CHINA

## Abstract

Consistent across time and cultures, men and male adolescents older than 14 years of age appear underrepresented in mood disorders, and are far less likely than women to seek psychological help. The much higher rate of suicide amongst males suggests that depression in men might be underreported. One of the core human motives is to seek acceptance by others and avoid rejection. Rejection Sensitivity (RS) has been conceptualized as the cognitive-affective processing disposition to anxiously expect, readily perceive, and intensely respond to cues of rejection in the behavior of others. RS has been previously linked with the onset and course of depression, but—as yet—has not been investigated longitudinally in a clinical population. We investigated the predictive role of RS to symptom deterioration 6 months after end-of- treatment in 72 male inpatients with depressive spectrum disorder. The BDI was administered at intake, end-of-treatment and 6 month follow-up. RS scores were obtained at intake. Rejection Sensitivity had additional predictive power on BDI scores at 6 months follow-up controlling for BDI scores at end-of-treatment (ΔR^2^ = .095). The results are discussed in terms of the importance of targeting RS during treatment, and highlight the fact that therapeutic follow-up care is paramount. Future research should investigate possible mediators of the RS–relapse-to-depression association, such as self-blame, rumination, neuroticism, pessimism, emotion dysregulation, and low self-esteem.

## Introduction

As a social species, one of our core motives is to seek acceptance and avoid rejection from others [1]. Accordingly, experiences of social rejection and exclusion have been found to affect people’s self-regulation [2–3], self-esteem [4] and social behavior by facilitating social withdrawal [3]. Interindividual differences in the cognitive-affective processing of experienced social rejection suggest a disposition of Rejection Sensitivity (RS; [5]). More precisely, RS has been conceptualized as the disposition to anxiously expect, readily perceive, and intensely respond to cues of rejection in the behavior of others [6], and is understood to be the combined result of individuals’ genetic predisposition and social learning [7]. Insensitive and rejecting relationships with the primary caregivers lead to the assumption that one will be rejected when seeking acceptance from significant others [8]. As a result, persons high in RS perceive potential signs of rejection more readily, and interpret ambiguous signs as negative, i.e., they interpret rejection where there possibly is none. These expectations lead to negative cognitions (such as self-blame), and negative affective reactions (anger, humiliation), which in turn provoke maladaptive behavior (aggression, social withdrawal, self-silencing) and subsequently the rejection of others, ultimately undermining significant relationships and mental health [9]. An increased vulnerability to depression after experiencing rejection further corroborates the predictions of this RS model [9–10].

RS not only is an important factor for understanding human social behavior in general, but also a risk factor for psychological ill health, in particular in vulnerable populations. Patients with depression often report lower self-esteem or feelings of worthlessness, and feel readily rejected [11]. Confirming this link between depression and RS, depressive symptoms have been shown to positively correlate with RS scores [12]. Interindividual differences in RS and hypervigilance for signs of rejection have been identified as predictors for depression [13]. People with depression, however, are not only more sensitive to possible signs of rejection; it is also plausible, that depressed individual’s behavior elicits rejection, for example by dysfunctional social communication behaviors such as excessive reassurance seeking [14], social withdrawal [13] and reduced eye contact [15]. Thus, the association between rejection and depression appears to be reciprocal [16], resembling a vicious cycle.

Even though large-scale epidemiological studies suggest an equal distribution of the frequency of mental disorders across the sexes [17–18], men are conspicuously underrepresented in mood disorders, a finding that is remarkably consistent across time and cultures [19]. Similarly, only half as many men than women seek psychological help [20]. Most compellingly, however, men commit suicide four times more frequently than women [21]. Depressed men often do not feel a connection to others, and believe that sharing their problems with others is a sign of weakness [22]. Men appear to express depressive symptoms differently, endorsing more withdrawal symptoms than women and tend to be more likely to report sex-role appropriate symptoms such as work problems and somatic concerns [23–24]. Qualitative studies show depressed men to report expectations of the male self as strong, successful, in control, capable of handling problems without help, and hiding emotions [22]. Until the age of 14 years, boys and girls are equally often diagnosed with depression [25], which may be an indication that the expression of depressive symptoms is culturally and developmentally influenced. Boys grow up in social contexts in which the suppression of emotions, relational hardening, defensive autonomy, and utilization of anger and aggression are the primary means of emotional expression [17]. According to the RS model, perceptions of rejection elicit cognitive-affective overreactions including hurt and anger. Acting these out renders them to be more likely to be rejected [26]. College men, who anxiously expect rejection, respond with heightened feelings of anger, hurt, and jealousy to hypothetical scenarios of partner rejection [27], and are described as jealous and controlling by their partners [5]. Men tend to utilize externalizing, outwardly directed response styles in coping with depressed mood [28–29]. Furthermore, rejection sensitivity (i.e. the anxious expectation of rejection) can facilitate male violence towards romantic partners [30]. For example, a study of husbands who killed their wives found that husbands’ rejection by their wives was the most frequent precipitant of the fatal incident [31]. These results in combination with the lack of research on men, depression and RS provide the rationale for the current studies’ focus on the role of RS in depressed men.

Relapse rates after the first depressive episode have been reported to be as high as 50–80% of cases [32]. Different factors have been identified moderating relapse rates, such as a high number of previous episodes, more residual depressive symptomatology, more daily hassles [33], a more avoidant way to deal with problems or a lower capacity to focus on positive matters (see [34] for a review). Rejection Sensitivity predicts the course and outcome of depression, as it has been shown to be associated with greater severity and duration of current major depressive episodes [35], increased propensity toward depression over time [36], and with poor psychosocial outcome [37–38]. Sensitivity to simulated social rejection is positively correlated with treatment outcome in major depression [39], suggesting outcome-related associations of RS, as depressed patients with higher RS benefited more from treatment, possibly as a function of the need to socially re-integrate [40]. The associations between RS, depression, and psychosocial outcome suggest a role of RS for relapse after a depressive episode. In patients with bipolar disorder type I, RS has been found to be associated with current self-ratings of depression and to predict increases in interviewer-rated depression scores at 6-month follow-up [41]. While responses to social exclusion have recently attracted growing research interest [42], studies on the specific role of RS in individuals with depression are still scarce, although such findings would be important for a better understanding of the factors and processes involved in symptom deterioration. As reported in a recent meta-analytical review [11], only four out of 21 available studies on RS investigated a clinical population, and none of these studies employed a longitudinal design. The few longitudinal studies currently available solely included healthy students. For example, in a study with healthy college students [43], RS did not predict later depression, but there was a reversed causal relationship in that depressed people showed higher RS later on. It has been argued that this could be due to the fact that social withdrawal behavior of depressed individuals can lead to repeated rejection by others and hence generate greater RS [11]. Rosenbach and Renneberg [11] also discuss the possibility that depressed people may fear to become stigmatized and rejected because of their mental health problems, which–in light of the results described previously–may be even more so the case for men. It has also been suggested, that the mutually reinforcing reciprocal influence between RS and depression is mediated by self-silencing, i.e., refraining from voicing one’s own emotions and needs, to avoid social conflicts [44]. It remains imperative, therefore, to identify the factors explaining the recurrence of depressive episodes so as to inform future clinical intervention strategies. In light of past research, we expect RS to have an impact on the likelihood of re-occurrence of depression. We hypothesize that RS scores assessed at the beginning of treatment predict self-reported depressive symptoms at six months follow-up while controlling for depressive symptoms at end-of-treatment follow-up in men.

## Methods and measures

### Participant characteristics

Recruiting started in January 2011, and ended in December 2013 in a hospital for clinical psychological interventions in Münster, Germany. Participants’ clinical diagnoses were determined using a structured interview for DSM-IV (SCID I, II) [45]. Exclusion criteria were a depression diagnosis with psychotic symptoms, comorbid schizophrenia and other psychotic disorders, comorbid substance- related disorders, or mental disorders due to a medical condition. 133 male in-patients with a diagnosis of depression were eligible for the study. Of these, data of 26 patients were incomplete at end-of-treatment. Of the remaining 107 patients, 35 had missing data at follow-up; therefore, 72 patients had complete data sets. Complete data sets included the following measures: The Beck’s Depression Inventory (BDI) [46] at intake, end-of-treatment and 6 month follow-up, and the Adult Rejection Sensitivity Questionnaire (ARSQ) [46] at intake and end-of-treatment.

Demographic characteristics and frequencies are displayed in Tables 1 & 2.

**Table 1:** Participant characteristics: Means and standard deviations.

	M	SD
Age	42.86	13.33
Treatment duration (days)	76.61	44.27
Number of mental disorders diagnosed per patient	2.61	1.26

**Table 2:** Participant characteristics: Frequencies.

	%
**Highest education level**	
Vocational schooling	15.3
Secondary School	19.4
College preparatory schools	20.8
University degree	38.9
Missing data	5.6
**Family status**	
married	40.3
married but separated	4.2
single	33.3
Living with partner	6.9
divorced	9.7
Missing data	5.6
**Diagnosis**	
recurrent Major Depressive Disorder	49.9
double depression	16.7
Major Depressive Disorder, single episode	29.2
Dysthymic Disorder	4.2
**Most frequent comorbidities**	
Personality Disorder	43.0
Anxiety Disorder	28.0
Attention Deficit Disorder	16.7
Eating Disorders	6.0
Obsessive Compulsive Disorders	8.0
Post-Traumatic-Stress Disorder	4.0
Somatization Disorder	6.0

### Measures

In addition, disorder-specific questionnaires were employed including the following:

Depression symptoms were assessed using the Beck’s Depression Inventory [46] (BDI). The BDI, a self-report measure to assess intensity of depressive symptoms and attitudes, is rated on a four-point scale. The scale has good psychometric properties with reported concurrent validity scores of .71 to .89 for different self-administered questionnaires testing depression, and test- retest reliability scores of .88. Cronbach’s alpha was calculated for the current sample with a value of alpha = .88, and is, therefore, comparable to other German samples [46].

Rejection Sensitivity was assessed using the ARSQ [47]. This instrument, initially developed by Downey & Feldman [5] to investigate attachment behavior and its relation to childhood maltreatment, consists of hypothetical social situations describing interpersonal interactions such as “You ask a friend to do you a big favor”. Each item is rated in regard to the concern or anxiety in the situation and the perceived likelihood that the other person would act in their favor. Rejection Sensitivity is then calculated by multiplying the level of rejection concern by the reverse of the level of acceptance expectancy. Hence, RS considers both, the anxiety and the perceived likelihood of rejection in interpersonal situations. The adapted German version [47] consists of two scales (anxiety and perceived likelihood of rejection) with 20 items each. It has good psychometric properties (high internal reliability: Cronbach’s alpha coefficient = 0.94 and test-retest reliability of rtt = 0.90). Cronbach’s alpha for the current sample was 0.95 for the anxiety subscale, and 0.93 for the rejection concern subscale.

### Procedure and study design

Self-report questionnaires (ARSQ, BDI) were administered during patients’ first week in hospital and at end-of-treatment. The diagnostic interviews (SCID I, II) also took place during the first seven days after admission, and were conducted by certified clinical psychologists. The treatment consisted of cognitive-behavioral treatment (CBT) with one-on-one sessions, group therapies and pharmacotherapy. Five months after end-of-treatment, each patient was contacted by a member of the quality control team, and asked to fill in the questionnaires again. Patients were kindly reminded after four weeks if they had not filled in the questionnaires at home. Thereafter patients were invited individually to the hospital to assess his/her wellbeing and to discuss the results of the self-report measures. Ethics approval was obtained from the Ethics committee of the “Medical Association Westfalen-Lippe” prior to any data collection, including all pseudonymized clinical routine data. All patients provided written consent that they data is used for research purposes.

### Statistical analysis

Statistical analyses were carried out using SPSS v.22. Paired samples- and independent t-tests, Pearson's correlations, an analysis of variance and regression analyses were performed. More precisely independent samples t-tests were performed to test the difference in depression severity for this with- and without follow-up data at intake and end of treatment. Paired samples t-tests were conducted to analyze pre-post differences in Rejection Sensitivity. Repeated measures ANOVA were conducted to analyze the difference between BDI at intake, end-of-treatment, and 6 month follow-up. For the regression analysis BDI scores at 6 month follow-up were entered as the outcome variable, and BDI scores at end-of-treatment were entered in the first-, ARSQ scores at intake in the second step.

## Results

To investigate possible selection bias, BDI scores were tested for significant differences between those with and without follow-up data at intake and end-of-treatment, respectively (72 with complete data sets vs. 35 with missing follow-up data). Independent samples t-tests revealed no significant difference in BDI scores t(105) = -.70; p = .49; d = 0.15) or RS scores (t(105) = .48;p = .63; d = 0.11) at intake or end-of-treatment (BDI: t(105) = -1.94; p = .056; d = 0.4; RS: t(100) = -.84; p = .403; d = 0.18) between those with or without follow-up data. Descriptive statistics for the final sample are presented in Table 3.

**Table 3:** Rejection sensitivity and depression scores.

	Intake		End-of-treatment		6 month follow-up	
	M	SD	M	SD	M	SD
ARSQ	11.56	6.20	9.75	5.35	-	-
BDI	20.71	9.68	8.50	7.54	12.24	11.43

ARSQ = Adult Rejection Sensitivity Questionnaire; BDI = Beck’s Depression Inventory

Rejection Sensitivity scores improved significantly after treatment (t(67) = 3.712; p < .001; d = 0.31). Repeated measures ANOVA were conducted to analyze the difference between BDI at intake, end-of-treatment, and 6 month follow-up. There was a significant difference between all three points in time (F(2) = 56,269, p < .001; pη^2^ = .442).

Pearson's corrrelations revealed no significant correlation between age or treatment duration respectively with BDI scores at discharge or at follow-up (Table 4).

**Table 4:** Pearson's correlations.

	BDI at discharge		BDI at 6 month follow-up
	r	p	r
Age	-.060	.614	-.117
Treatment duration	.181	.129	.220

r = two tailed; Treatment duration refers to days of treatment.

**Fig 1:**
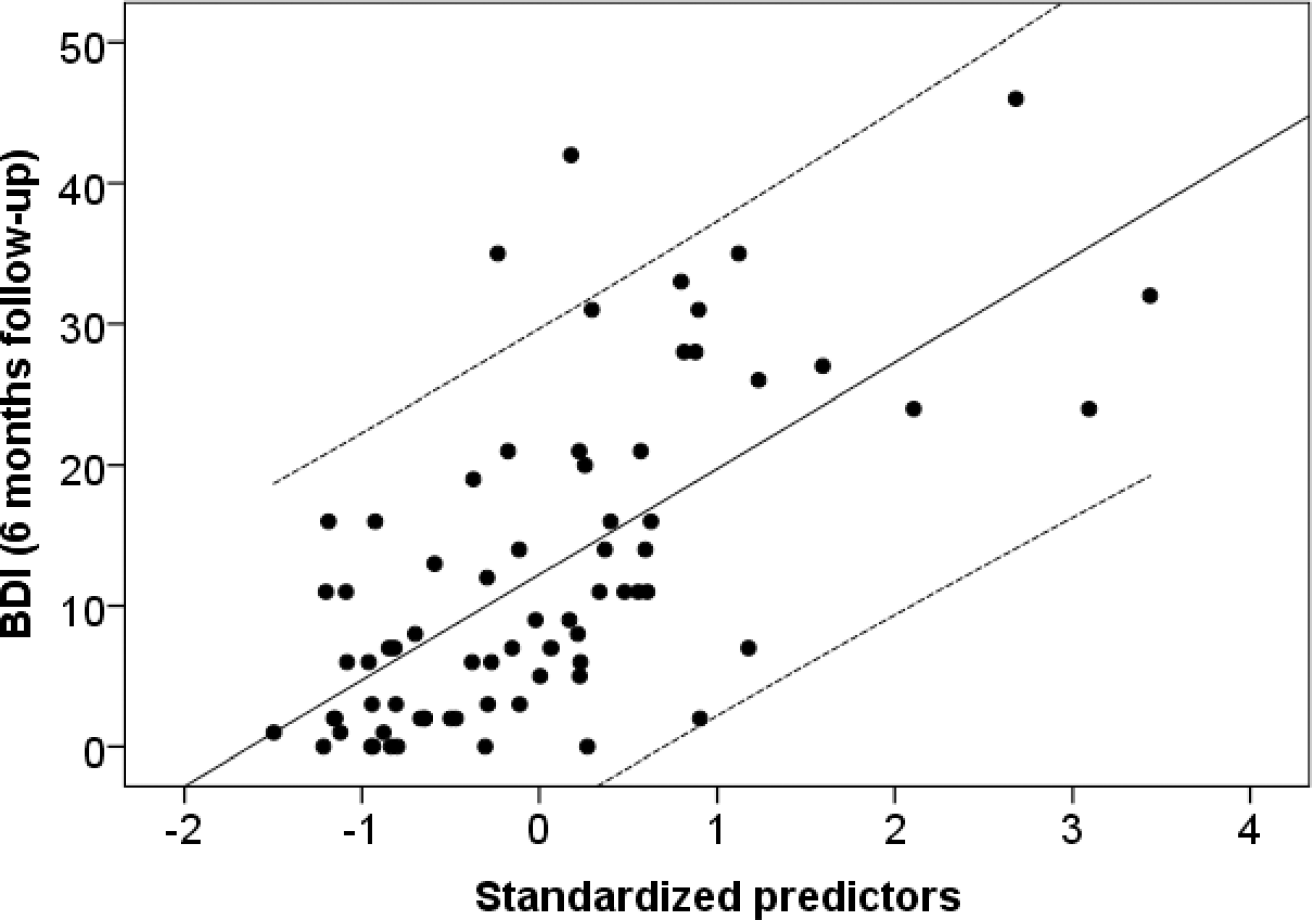
Rejection sensitivtiy predicts depressive symptom deterioration. Dependent variable: BDI at 6 month follow up; Dashed lines = CI (95%). In order to rule out the possible influences of a comorbid PD diagnosis, we repeated the analyses without patients with a PD and found the results to remain unchanged (ΔR^2^ = .105; β = .325; t = 2.616; p = .013; R2 = .417; R^2^corr. = .386; F(2) = 13.57, p < .001).

For the main analysis, a multiple regression analysis was conducted to test for the additional predictive power of RS at intake on BDI scores at 6 months follow-up, while controlling for BDI scores at end-of-treatment. BDI scores at end-of-treatment were entered in the first step, RS scores at intake in the second step, predicting the outcome variable BDI score at 6 month follow-up. BDI at end-of-treatment predicted BDI at 6 month follow-up (β = .580; t = 5.961; p < .001). Entering RS at intake in the second step improved the model significantly (ΔR^2^ = .095; β = .320; t = 3.397; p < .001). The total model explained 41.5% of the variance at 6 month follow-up BDI (R2 = .432; R^2^corr. = .415; F(2) = 26.21, p < .001) (Fig 1).

## Discussion

We investigated the predictive role of RS at intake for depressive symptom deterioration in men six months after inpatient treatment controlling for depression scores at the end of treatment. We expected for RS to predict depression scores 6 months after treatment while controlling for post treatment depression scores. The results show that RS independently predicted depressive residuals, suggesting that RS is an important factor, affecting outcome after in-patient treatment and, therefore, potential relapse after depression.

The current findings are in line with results reported by Ng & Johnson [41], who found in a sample of patients with bipolar disorder type I that Rejection Sensitivity at baseline predicted increases in depression, but not mania, over the following six months. In their research, heightened RS was also correlated with poorer quality of life, social support, and psychological wellbeing. These findings highlight the importance of psychological follow-up care. While patients benefited at symptom level from the therapeutic treatment as indicated by improved BDI scores at end-of-treatment, the baseline level of RS was a predictor for the extent of increases in BDI scores between end-of-treatment and follow-up. These results suggest that patients high in RS at intake may be at increased risk for relapse after depression, even though benefiting from treatment initially. Psychological treatment of depression should, therefore, strengthen patients’ social competencies, and improve their emotion regulation skills when faced with social exclusion. This is all the more pertinent as patients with depression are more prone to experience social exclusion, either initiated by their own behavior as outlined above, or by being stigmatized as “mental patients”.

While previous research has conceptualized RS as a stable trait [5], in the present study RS scores decreased after treatment suggesting that RS is amenable to change. Future research should investigate whether the risk of relapse after depression treatment could be significantly reduced over the long-term if addressing RS during intervention and follow-up care. As RS seems to affect depressive residuals independent of BDI at end-of-treatment, it might be important to control for RS in intervention trials, as RS may differentially affect treatment outcome.

RS has been conceptualized as the disposition to anxiously expect, readily perceive, and intensely respond to cues of rejection in the behavior of others [6]. In accordance with the model, we suggest that the mechanisms at play here, rendering highly RS men more vulnerable to depression after treatment are a) their oversensitivity to rejection cues, b) their propensity to interpret ambiguous signs as negative, and c) the resulting, negative cognitions and affective reactions that give rise to maladaptive behavior which in turn provoke the rejection by others and undermine significant relationships and mental health [9]. More precisely, highly RS men may more readily use self-blame, may feel humiliated or angry more readily, revert to social withdrawal or aggression, which makes them more readily rejected by others and further enliven the rejection-depression slope.

Other factors that may mediate the association between RS and depressive symptom deterioration should be considered in future research. One such factor could be rumination. RS not only encompasses anxious expectation of rejection experiences, but also the propensity of affected individuals to dwell on, i.e., to ruminate about the rejection experience, thereby increasing their sensitivity to future rejection even more. This is supported by results showing that RS predicts rumination after 6 months controlling for baseline rumination, depression and gender [48]. Another intriguing possibility—yet to be examined—is that RS may generate the very stressors that lead to subsequent depression. According to the stress generation hypothesis [49–50], depression-prone individuals, tend to experience higher rates of life stressors that are at least partially influenced by their own behavioral and cognitive characteristics (i.e., dependent stressors), but do not differ in the prospective occurrence of stressors that are outside their control (i.e., independent stressors). Consistent support for the view, that RS exerts a deleterious effect on interpersonal relationships through self-fulfilling behavioral tendencies has been forwarded by Downey and Feldman [5], as individuals high in RS tend to experience greater dissatisfaction in their relationship, react more negatively to ambiguous behavior in others and are more likely to experience a breakup in their romantic relationships. Furthermore, the negative behavior (e.g., negative voice tone, denying responsibility for problems in the relationship, and putting down their partner) of individuals high in RS during conflict-related discussions within observational settings has been associated with negative post-discussion affect in their romantic partner [51].

Men express depression differently to women [17], employing to a greater degree both overt (physical, verbal, nonverbal aggression) and covert (withdrawal, avoidance, self-silencing) negative coping strategies, that ultimately undermine their significant relationships and their mental health [9;52]. Anger is one of the few socially accepted emotions [53]. We would argue that this externalizing behavior more readily leads to actual rejection and, therefore, also to more RS. Future research should investigate this line of reasoning. Moreover, Harper and colleagues [44] outline that the depression- RS association is mediated by behaviors that are characterized by holding back one's own emotions aiming at avoiding interpersonal conflicts (so called self-silencing). It has been argued, therefore, that those high in RS and a tendency to not voice their own needs because of their fear of social conflicts are especially prone to depressive symptoms. In contrast, supporting social relationships reduce the extent of RS in people with depressive and anxiety symptoms [54].

Even though the present findings provide support for the hypothesis that RS predicts depressive symptom deterioration, the results need to be interpreted with caution for three reasons. Firstly, as RS was assessed using self-report measures, the RS-depression correlation (both self-report measures) may have occurred as a function of common method bias. Previous research has outlined significant effects of RS on self-rated, but not interviewer-rated depression [55]. This could be due to a tendency of individuals with depression to evaluate themselves more harshly. Secondly, even though the present results revealed no differences in BDI scores between those with and without follow-up data, there was a trend indicating that those not completing the self-report questionnaires at follow-up had more severe symptoms of depression. This may limit the generalizability of the results. Thirdly, the causality of effects cannot be disentangled in the current study. Along the hen- or egg analogy, it can be argued, that those individuals who had experienced more severe depressive symptoms may develop a greater sensitivity to rejection because of being confronted with more mental illness stigma. Ideally, future studies should assess premorbid levels of RS in patients at risk for depression, analyze the temporal stability of RS during and after clinical treatment and use a longitudinal assessment in order to investigate how baseline RS levels influences outcomes and relapse rates over time. Future research should address the current lack in investigating other, potentially relevant variables that may interact with RS in predicting depression, such as self-blame [12], rumination [48], neuroticism [5], pessimism [56], emotion dysregulation [57], and low self-esteem [5].

## Conclusion

The current study identified RS as a significant, independent predictor for depressive symptom deterioration in men, after end-of-treatment. This result highlights the importance of therapeutic strategies targeting RS, and underscores the paramount importance of therapeutic follow-up care. Future research should investigate possible mediators of this RS-relapse to depression link, such as self-blame, rumination, neuroticism, pessimism, emotion dysregulation, and low self-esteem.

## Supporting information

S1 File The data file. (SAV)
